# Using Young Mothers’ Clubs to Improve Knowledge of Postpartum Hemorrhage and Family Planning in Informal Settlements in Nairobi, Kenya

**DOI:** 10.1007/s10900-014-9986-8

**Published:** 2015-01-14

**Authors:** Gathari Ndirangu, Anthony Gichangi, Lynn Kanyuuru, Jane Otai, Rose Mulindi, Pamela Lynam, Nancy Koskei, Hannah Tappis, Linda Archer

**Affiliations:** 1 Jhpiego Kenya, 2nd Floor, Arlington Block, 14 Riverside, P.O. Box 66119–00800, Nairobi, Kenya; 2 Jhpiego/USA, An Affiliate of Johns Hopkins University, 1615 Thames St., Baltimore, MD 21231 USA

**Keywords:** Urban health, Health promotion, Maternal health, Postpartum hemorrhage, Family planning, Kenya, Slum

## Abstract

Women living in Nairobi’s informal settlements face a higher risk of maternal death than those living elsewhere in the country, and have limited knowledge of actions they can take to improve their chances of survival during pregnancy and childbirth. As one strategy to reach this high risk group, Jhpiego has implemented young mothers’ clubs (YMCs). These clubs comprise mothers aged 18–30 who come together on a weekly basis to share experiences and solutions to their challenges while receiving health education from health facility staff and community health workers (CHWs). The aim of this study was to assess whether the YMC strategy could be used to improve participants’ knowledge of postpartum hemorrhage (PPH), positive behavior around childbirth, and family planning. Participants in nine YMCs (n = 193) across four informal settlements were interviewed to assess their knowledge of safe motherhood topics before and after a series of eight health education sessions. Data were analyzed with the McNemar test to determine significance of change in knowledge pre- and post-intervention. The largest improvements were observed in knowledge about what to include in a birth plan, with correct responses increasing from 32 to 73 % (*p* < 0.001), 58–93 % (*p* < 0.001), 36–66 % (*p* < 0.001), 58–85 % (*p* < 0.001), and 64–88 % (*p* < 0.001) for identifying a birth companion, budget, skilled birth attendant, emergency supplies, and place of birth, respectively. Less substantial improvements were observed in knowledge of danger signs of PPH (up 10 % from 77 %, *p* = 0.003). Although knowledge of actions to take in the event of bleeding after delivery did significantly improve, final knowledge scores remained low—knowledge to urinate increased from 14 to 28 % (*p* < 0.001) and to breastfeed from 12 to 24 % (*p* = 0.005). Even though the vast majority of respondents (84 %) knew before the intervention that a woman should space pregnancy by at least 2 years after delivery, there was an increase to 94 % after the sessions (*p* = 0.008). Overall, participants demonstrated significant improvements in knowledge of safe motherhood and family planning topics, suggesting that the materials and methods used were generally effective for improving knowledge among this high risk group.

## Introduction

The target of United Nations Millennium Development Goal (MDG) 5 is to improve maternal health through reduction of the maternal mortality ratio (MMR) by three-quarters between 1990 and 2015. Although there has been a 45 % global decline in the MMR between 1990 and 2013, an unacceptably high number of maternal deaths of 814 per day continues to occur worldwide [1]. In many countries the annual average rate of decline in maternal mortality is inadequate to achieve the desired 75 % reduction by 2015 [2]. In Kenya, MMR has remained high at 488 deaths per 100,000 live births [3], placing it as the country with the eighth highest MMR in the world [2]. Globally, hemorrhage is the leading direct cause of maternal death, being responsible for 27 % of maternal deaths between 2003 and 2009 [2]. In sub-Saharan Africa, 25 % of maternal deaths during the same period resulted from hemorrhage. In the majority of these deaths (15 %), the bleeding occurred during the postpartum period [4]. The causes of maternal death in Kenya are estimated to mirror the pattern in sub-Saharan Africa.

Rapid urbanization, fueled by high levels of rural-to-urban migration under conditions of poor economic performance, has led to the high growth of urban informal settlements in many African countries. These informal settlements are characterized by high population, poor housing, lack of basic amenities, and low availability and utilization of formal health services. Like many other health indicators, maternal mortality is highest among those living in such conditions. In Nairobi’s informal settlements, the MMR was estimated to be 706 per 100,000 live births in 2009 as compared to a national MMR of 488 per 100,000 live births [3, 5]. Finding effective ways to provide health care in limited resource settings such as these is becoming an issue of increasing urgency.

Understanding of maternal mortality, and by extension postpartum hemorrhage (PPH), is limited among the urban poor, and failure to correctly and promptly identify danger signs has been identified as one of the barriers to accessing formal emergency obstetric services in the informal settlements of Nairobi [6]. Studies also show that 50 % of women in Nairobi’s urban slum communities resume sexual relations by the3 month, but relatively few initiate contraceptive use during the first 6 months postpartum [7].

Jhpiego has been working in the Nairobi informal settlements to improve health seeking behaviors, water treatment, hygiene, and sanitation. Since 2005, intense focus has been placed on access to antenatal care, HIV counseling and testing, screening for cervical cancer, and management of sexual gender-based violence. One of the strategies employed by Jhpiego has been the organization and facilitation of young mothers’ clubs (YMCs). Through these clubs, mothers aged 18–30 come together on a weekly basis to share experiences and solutions to their challenges while receiving health education from health facility staff and community health workers (CHWs). Depending on the concerns raised, mothers are then referred to various institutions including health facilities for further management. YMCs are based on the notion that mobilizing communities around a health concern and fostering community participation can increase awareness of important issues and promote ownership of interventions, thereby facilitating positive behavior change and adherence [8].

This study was designed to assess whether YMCs could be utilized to communicate safe motherhood and family planning messages to young mothers in these settlements, with a view to improving their knowledge of PPH, positive behavior around childbirth, and family planning. Specifically, this study was designed with the programmatic aim of testing whether the materials and methods developed to communicate information about the risks of PPH and the relationship between PPH and family planning would result in improved knowledge among YMC participants.

## Methods

**Fig. 1:**
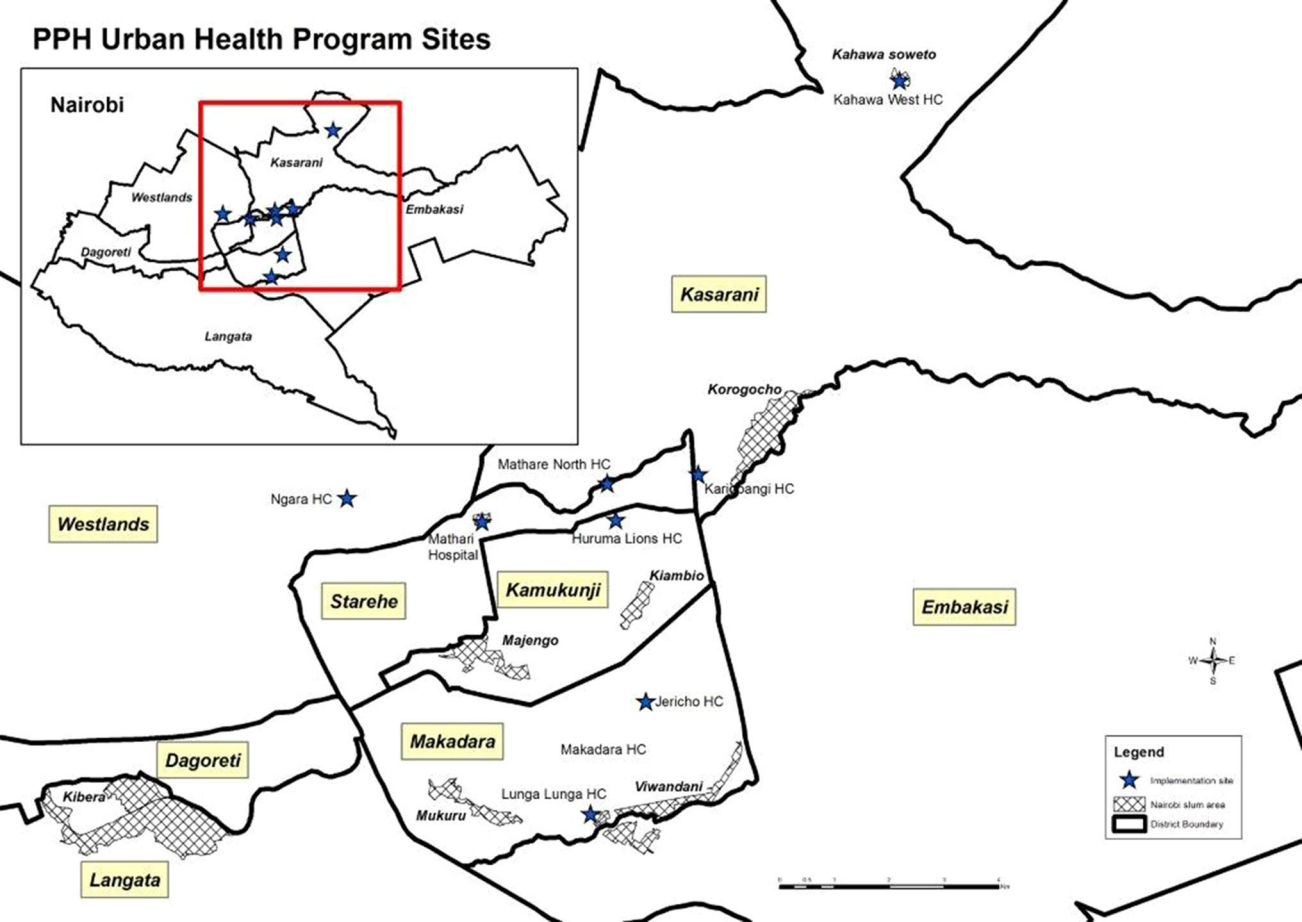
Map of Nairobi with location of study sites

The study was conducted in nine health care facilities that serve populations living in four informal settlements where Jhpiego has been providing technical support for ongoing maternal and child health programming: Mathare, Korogocho, Viwandani, and Kahawa Soweto. These facilities, illustrated in Fig. 1, were purposively selected based on their location within—or serving the population of—an informal settlement, provision of maternity services, current registration with the Ministry of Health, and willingness to participate in the study.

The target population was young mothers living in the four informal settlements: the 2009 census count for these areas was almost 99,000 people. The study population was mothers less than 30 years of age living in the catchment areas of the nine health facilities. The women were also required to have given birth within the last 2 years or be pregnant at the time of the study, and willing and able to participate in the YMCs. Based on past experience implementing YMCs in Viwandani and Korogocho settlements, the maximum group size was set at 30 participants, and organized gatherings were limited to eight sessions of no more than 3 h each held at the health facility. Group members were encouraged to continue meeting independent of the organized sessions, but the YMC curriculum, CHW involvement, and study period were limited to the eight sessions at each facility. CHWs working in the catchment areas of the nine facilities identified mothers that met study inclusion criteria and invited them to participate in the YMC. Mothers below age 18 were allowed to participate in the YMC, but were not included as study participants.

Four service providers from each of the nine facilities were trained to improve their ability to recognize PPH in mothers who presented with symptoms or who already manifested PPH. The trained service providers then cascaded the training to other health workers within the maternal and child health units at their facilities. The trained service providers further cascaded relevant parts of the training to CHWs to ensure that they were able to recognize and refer mothers suffering from PPH to the facility, and to counsel pregnant women in the community on the dangers of PPH and how community members can help ensure that women with PPH receive lifesaving care.

On the first day of the YMC, the participants were informed about the study, an oral consent form was read to them, and their verbal consent was obtained to participate in the study. The CHW then administered a quiz to assess participants’ knowledge on PPH and family planning as well as current use of family planning methods. This quiz was administered either orally or self-administered depending upon the literacy level of the club member. A Swahili version of the questionnaire was used for participants who could not read or speak english. Each participant was given a unique study number that was used throughout the YMC.

The YMCs met on agreed-upon afternoons once per week for discussions led by the CHW, with support from CHW supervisors and health facility staff on certain topic areas. Each training session focused on one of the following topics: family planning, preconception care, nutrition, focused antenatal care, PPH, infection prevention, postnatal care, and postpartum family planning. Session content was based on materials used in previous USAID- and private donor-supported health programs, with the exception of the PPH content, which was developed in consultation with the Ministry of Public Health’s Department of Reproductive Health. At each session, the CHW and service providers presented information in an interactive manner, with the curriculum serving as a starting point for questions from participants and discussion of shared experiences. At the final session, the same quiz administered at the first session was repeated.

The completed questionnaires were collected and secured in sealed envelopes in a locked cupboard in the facility until they were collected by the study team during routine visits to the facilities. The list linking the unique study number to participant names was kept separately until the post-tests were administered. It was only used when a study participant forgot her study number. The data were entered into a password-protected database at the Jhpiego Nairobi office, and the questionnaires were stored in a secure, locked cabinet throughout the intervention period. Participant responses on the knowledge of PPH and family planning were scored and the proportions of participants who responded correctly were linked to allow comparison of pre- and post-intervention results. Data were analyzed using STATA 12. Significance of changes was tested using the McNemar test because we were interested in determining whether the proportion of correct responses varied for matched pairs at pre- and post-intervention.

### Ethical Considerations

The study protocol was reviewed and determined not to be human subject research. The protocol was approved by the ethical review boards of the Johns Hopkins Bloomberg School of Public Health and the Kenya Medical Research Institute.

## Results

As shown in Table 1, a total of 256 women participated in the first meeting of YMCs in the nine health centers that were selected for the study, 84 % of these women reported giving birth to their last child in a health facility. Of these 256 mothers, 75 % (193) completed both the pre- and post-orientation questionnaires, which are the source of the majority of our comparisons. The loss between pre- and post-testing ranged from 0 to 53 % across the nine sites over the 8 week period. The average number of women who attended the YMC sessions ranged from 21.4–28.4 across the facilities (or 26.0 for all nine facilities).

**Table 1:** Characteristics of study population, by closest health facility

Informal settlement	Korogocho and Kahawa Soweto			Viwandani and Kiambio			Mathare			Total
Health centers/clinics	Kahawa West	Kariobangi	Mathare North	Jericho	Lunga Lunga	Makadara	Huruma Lions	Mathari	Ngara	
YMC participants at first session	30	30	28	29	27	29	27	26	30	256
Delivered last child at a clinic or health facility	89 %	95 %*	88 %	100 %	89 %	100 %	52 %	55 %	100 %	84 %
Average attendance at YMC	27.0	26.2	26.0	27.6	28.4**	27.6	24.0	21.4	25.4	26.0
Participated in both pre- and post-intervention interviews	24	24	19	17	26	29	23	17	14	193

* Pre-intervention interviews missing; this is from the post-intervention interviews

** Young mothers under age 18 may have attended some YMC sessions, but did not participate in pre- and post-intervention interviews

Table 2 presents women’s knowledge of safe motherhood topics before and after participation in the YMC. The largest absolute improvements (of up to 41 % points) were observed in knowledge about what to include in a birth plan. Participants’ awareness that a birth plan should identify where to give birth, identify a skilled attendant, include plans on how to get to the facility, include how to save money, identify a birth companion, and put together items for a clean and safe birth increased significantly from the first to last YMC sessions.

**Table 2:** Safe motherhood knowledge of YMC participants (n = 193)

	Pre	Post	Change	*p* value*
	% (n)	% (n)		
Know the minimum number of antenatal care visits that a pregnant woman should make	89 (172)	96 (185)	7	**0.015**
Know a birth plan should identify where to give birth	64 (123)	88 (170)	24	**<0.001**
Know a birth plan should identify a skilled attendant	36 (69)	66 (128)	30	**<0.001**
Know a birth plan should include plans on how to get to the facility	66 (128)	79 (153)	13	**0.005**
Know a birth plan should include how to save money for care and delivery	58 (111)	93 (179)	35	**<0.001**
Know a birth plan should identify a birth companion	32 (61)	73 (140)	41	**<0.001**
Know a birth plan should put together items for a clean and safe birth	58 (112)	85 (165)	27	**<0.001**
Correctly identify bleeding, anemia, and swelling of the face, hands, and feet as danger signs during pregnancy	77 (149)	87 (168)	10	**0.003**
Correctly identify excessive bleeding as a danger sign after delivery	89 (172)	94 (181)	5	0.163
Know to breastfeed if there is too much bleeding after delivery	12 (24)	24 (47)	12	**0.005**
Know to urinate if there is too much bleeding after delivery	14 (27)	28 (55)	14	**<0.001**
Know to seek care at the hospital as fast as possible if there is excessive bleeding after delivery	85 (165)	94 (182)	9	**0.006**

The *p* values presented in bold represent acquisition of knowledge at or above 95 % significance level

* McNemar test

Less substantial improvements were observed in knowledge of danger signs and actions to take in the event of bleeding after delivery. The majority of participants could correctly identify obstetric danger signs at baseline. However, the proportion of participants who could identify vaginal bleeding, anemia, and body swelling as danger signs during pregnancy still significantly increased from 77 % at the first YMC session to 87 % after the last session (*p* = 0.003). The proportion of participants who correctly identified bleeding after childbirth as a danger sign was high at the beginning, and also increased, although not significantly, from 89 % at the first session to 94 % after the last session (*p* = 0.163). Similarly, the proportion of participants who knew to seek care at the hospital as fast as possible in the event of excessive bleeding after delivery increased from 85 to 94 % (*p* = 0.006).

In contrast, only a small proportion of participants demonstrated baseline knowledge of other actions to take if there is too much bleeding after delivery at baseline. Although the proportion of participants who knew to encourage a woman to breastfeed and the proportion who knew to encourage a woman to urinate in the event of heavy bleeding after childbirth both doubled over the course of the intervention, less than 30 % of participants demonstrated knowledge of these actions after the last YMC session.

Table 3 presents women’s knowledge about postpartum family planning before and after participation in the YMC. Even though the vast majority of respondents (84 %) knew before participating in the YMC that a woman should space pregnancy by at least 2 years after delivery, there was a statistically significant increase of 10 % points between the pre- and post-test (*p* = 0.008). Before the intervention, only 18 % of the women knew the three requirements for lactational amenorrhea to act as a contraceptive method; this increased to 52 % following the intervention (*p* < 0.001). In contrast to all other knowledge variables, there was a statistically significant decline in the proportion of young mothers who knew that a woman can start using family planning immediately after birth (from 57 to 46 %) from pre- to post-intervention; this was the only negative change observed.

**Table 3:** Family planning knowledge of YMC participants (n = 193)

	Pre	Post	Change	*p* value*
	% (n)	% (n)		
Know a woman should wait at least 2 years before getting pregnant again after delivery	84 (162)	94 (182)	10	**0.008**
Know a woman can start using family planning immediately after delivery	57 (110)	46 (88)	−11	**0.006**
Know the three elements of LAM if it is to be used as a method of family planning (baby must be less than 6 months old, must be practicing exclusive breastfeeding, and periods cannot have returned)	18 (35)	52 (100)	34	**<0.001**

The *p* values presented in bold represent acquisition of knowledge at or above 95 % significance level

* McNemar test

Finally, the proportion of young mothers reporting current use of a family planning method rose from 77 to 86 % (<0.001). There was no significant change, however, in the family planning method mix used pre- and post-intervention; the method mix stayed predominantly injectables (45 % pre and 42 % post), implants (24 % pre, 25 % post), and the pill (19, 18 %). (Data are not shown.)

## Discussion

Overall, the YMC participants demonstrated improvements in knowledge of safe motherhood and family planning topics over the course of the study, suggesting that the materials and methods used were generally effective for improving knowledge among the study population. Studies of similar interventions to raise awareness of postabortion care and family planning found similar results, suggesting that YMCs are a viable mechanism for education and promotion of maternal health service utilization among residents of Nairobi’s informal settlements [9].

Evidence suggests that raising awareness of obstetric danger signs can improve birth preparedness, promote skilled birth attendance, and reduce delays in care seeking during obstetric emergencies [10, 11]. A study on community participation in prevention of PPH carried out in Nigeria suggested that even in circumstances where only modest levels of participation can realistically be achieved, community mobilization can have a significant impact on the uptake of a potentially lifesaving health intervention, and in turn, can help promote policy change [8]. The use of women’s groups for participatory learning and action has been shown to be a cost-effective strategy to improve maternal and neonatal survival in low-resource settings [12].

The fact that greater improvements in knowledge were observed in general aspects of birth preparedness planning than in specific actions to take in the event of PPH suggests that revision of the YMC curriculum to include more time dedicated to these specific actions and more implementation research to refine messaging on these issues should be considered in any replication or scale-up of YMC for maternal health promotion. It is possible that the women failed to understand how simple physiological actions such as urinating and breastfeeding could be used to manage heavy bleeding after childbirth. Revising the curriculum to explain the effectiveness of these specific actions and emphasize the importance of performing these actions may improve retention of knowledge. Reviewing successful birth preparedness education models used in other settings, such as the Philani Plus “mentor mother” home visit program implemented in South African townships [13] or the family health education model implemented by the Maternal and Newborn Health in Ethiopia Partnership [14], could also be done.

Above all else, findings from this study also point to the need for increased investments in community health education efforts in Nairobi’s informal settlements, especially with regard to the management of obstetric complications and requirements for effective postpartum family planning. The low levels of knowledge documented among these women, many of whom delivered their last child at a health facility or have had some contact with the public health system (as indicated by their participation in YMC), is sufficient indication of the need for renewed attention in these areas; and it is fair to assume that women who have no contact with the health system have even less knowledge.

The Ministry of Health has been scaling up its Community Health Strategy, which is anchored on the use of CHWs to promote skilled care during pregnancy, childbirth, and in the postnatal period [15]. CHWs receive a 7 day training based on a national curriculum and are then tasked to make regular contact with women in their catchment areas to encourage them to seek skilled care from the formal health sector and promote the use of family planning; however, only 1 day of the training is dedicated to maternal health topics. Renewed focus on raising awareness on the value of family planning through the media, CHWs, and health workers may have been responsible for a high knowledge of family planning before the intervention. In addition, the increase in knowledge after the intervention despite this high baseline level suggests that the curriculum was effective in this aspect. Nonetheless, the Ministry of Health will need to strengthen the CHW training curriculum to place more emphasis on other aspects of maternal health such as the need for recognition of obstetric complications and prompt management by skilled attendants, as well as postpartum family planning. Additional training on how to conduct group health education sessions and other outreach activities would help CHWs improve their ability to convey health messages to adult populations.

The decline in the proportion of young mothers who knew that a woman can start using family planning immediately after childbirth could have resulted from a misinterpretation of the information provided about the use of the lactational amenorrhea method (LAM). Women may have thought that they should not use any modern family planning method other than LAM. It is also possible that health care providers may not have emphasized that LAM is a modern family planning method; therefore, the young mothers could have responded using this misunderstanding during the post-test quiz.

A limitation of this study is the lack of socio-demographic information about YMC participants. Collecting this information would have allowed an assessment of variation in YMC participation or knowledge retention by age, parity, education level, relative wealth status, and other individual and household-level characteristics. It is also important to remember that results only reflect changes among young mothers who participated in both the pre- and post-intervention assessments. It is possible that results would differ if all women participating in YMCs were interviewed and their level of participation or attendance taken into account. External validity of study findings is also limited by the fact that YMC sessions were held at health facilities. Knowledge of women who agree to participate in activities at a health facility may differ from that of women not approached or not interested in participation in sessions at a health facility, and may have resulted in some of the young mothers feeling that they should answer the behavior questions (delivery of last and next child, and family planning acceptance) in a certain manner to gain approval of the health facility staff. More broadly, the characteristics of women and factors affecting implementation of interventions in Nairobi’s informal settlements may differ substantially from those of urban poor elsewhere in the world. Despite these limitations, these study findings contribute evidence that can be used to inform strategies for reducing health inequities and improving maternal health outcomes in Nairobi’s urban settlements.

## Conclusions

Our study demonstrates that the use of YMCs can be an effective strategy for improving knowledge and influencing health seeking behavior and intention to use contraception among women living in informal settlements. By providing correct information about skilled health care and family planning, these clubs provide excellent opportunities to improve the recognition of obstetric complications; improve knowledge on when and where to seek emergency skilled care for these complications; enable women to make correct decisions to have skilled care during pregnancy, childbirth, and in the postnatal period; and increase the uptake of family planning services, thereby improving maternal and newborn health outcomes among the urban poor. Findings from our study provide a platform upon which the Ministry of Health can strengthen its Community Health Strategy by revising the CHW training curriculum to include vital information that will help prevent maternal deaths (i.e., information about PPH, positive behavior around childbirth, and family planning).
